# Body-weight support is the primary driver of elevated walking cost in cerebral palsy

**DOI:** 10.21203/rs.3.rs-8832830/v1

**Published:** 2026-02-16

**Authors:** Andrew J. Ries, Katherine M. Steele, J. Maxwell Donelan, Michael H. Schwartz

**Affiliations:** Gillette Children’s Specialty Healthcare; University of Washington; Simon Fraser University; Gillette Children’s Specialty Healthcare

**Keywords:** Cerebral palsy, walking energetics, metabolic cost, body-weight support, lateral stabilization, gait biomechanics, rehabilitation engineering, assistive technology

## Abstract

**Background:**

Children with cerebral palsy (CP) exhibit substantially elevated energetic costs of walking, yet the biomechanical origins of this excessive cost remain unclear, limiting the effectiveness of current interventions.

**Methods:**

We tested the hypothesis that elevated energetic cost of walking in CP arises primarily from increased demands for body-weight support and lateral stabilization. Using a custom mechatronic system, we independently applied controlled vertical body-weight support (1–60% body weight) and mediolateral stabilization stiffness (50–1500 N/m) while children with CP and typically developing (TD) peers walked on a treadmill at a fixed nondimensional speed. We quantified steady-state energetic cost using indirect calorimetry and used linear regression models to determine energetic responses to each intervention.

**Results:**

Providing body-weight support significantly reduced net energetic cost in both groups, with a 3.5-fold greater effect in children with CP (n = 23) compared to TD peers (n = 10). Across the 1–60% support range, energetic cost decreased by 41% in CP, normalizing walking energy expenditure to TD levels. Higher baseline energetic cost of walking and greater knee flexion during stance were associated with larger energetic reductions (p < 0.01). In contrast, mediolateral stabilization produced negligible energetic effects in both CP (n = 14) and TD groups, with no demographic or biomechanical predictors of response.

**Conclusions:**

Body-weight support is the dominant contributor to elevated energetic cost of walking in children with CP, whereas lateral stabilization contributes minimally under the conditions tested. These findings identify gravitational support as a key biomechanical target for energy-focused rehabilitation and assistive technology interventions.

## One sentence summary

Providing body-weight support reduced the energetic cost of walking in children with cerebral palsy to levels similar to those of typically developing peers.

## Background

Cerebral palsy (CP) is the most common cause of childhood-onset motor disability, affecting over 750,000 people in the United States alone^1–3^. Approximately two-thirds of these individuals are able to walk independently or with assistive devices^4^. However, despite this ambulatory capacity, walking for people with CP is energetically demanding, often requiring more than twice the metabolic energy of typically developing (TD) peers^5–12^. This elevated energetic cost of walking is comparable to jogging or stair climbing, and is associated with reduced activity levels and diminished quality of life^13^.

Although numerous interventions aim to improve gait in CP, the biomechanical mechanisms underlying this excessive energetic cost remain poorly understood, limiting our ability to treat it. Conventional interventions such as orthopedic surgery, botulinum toxin injections, and selective dorsal rhizotomy aim to improve gait quality but don’t consistently reduce the energetic cost of walking^14–18^. In addition, spasticity, long assumed to be a primary driver of the excessive energetic cost of walking, has been shown to contribute minimally^15,19–21^. Together, these realizations underscore a critical gap in our understanding of the biomechanical and physiological drivers that contribute to elevated energetic costs and the interventions designed to reduce them.

In contrast with CP, the energetics of walking for TD individuals has been extensively studied. Experimental and theoretical analyses have shown that supporting and stabilizing the body against gravity typically accounts for less than 10% of the total energetic cost of walking^22–33^. Support cost is kept low through well-aligned joints that allow the legs to act like inverted pendula requiring relatively small muscle forces to support the body^34^. Stabilization cost is kept low by maintaining balance over a narrow base-of-support requiring only small adjustments to the lateral foot placement from step to step to maintain stability^25^. Although these tasks represent a relatively minor share of the total energetic cost of walking in TD individuals, they have nonetheless inspired targeted interventions, such as exoskeletons, that have successfully reduced this 10% cost^35–39^. This illustrates how a thorough understanding of the underlying biomechanical mechanisms of walking energetics can inform effective interventions.

Current evidence suggests that the biomechanical efficiencies observed for TD walking do not fully extend to CP gait^40^. Prior work has shown that individuals with CP perform ~ 25% more mechanical work during walking, yet exhibit nearly 100% higher energetic cost of walking compared to matched TD individuals^40^. This discrepancy between CP and TD energetics persists across a wide range of walking speeds, implicating tasks that are relatively insensitive to walking speed, such as supporting the body against gravity and stabilizing the center of mass, as potential contributors to the elevated energetic cost of walking in CP^41–45^. Common gait deviations observed in CP, including excessive joint flexion and increased step width, may increase energetic costs by impairing efficient body-weight support and stability mechanisms, respectively. For example, walking with excessive knee and hip flexion (i.e., crouch gait) increases muscle activity required to support the center of mass against gravity, reducing reliance on passive skeletal alignment and therefore elevating energetic cost^34^. Similarly, walking with increased step width amplifies the mechanical work needed to redirect the body from step to step, elevating the energetic cost of walking^43^.

Based on this evidence, we hypothesized that the elevated energetic cost of walking in CP arises primarily from increased demands for body-weight support and lateral stabilization. To test this hypothesis, we developed a custom mechatronic system capable of independently applying controlled vertical body-weight support and mediolateral stabilization forces during treadmill walking for CP and TD children^46–51^. This system allowed us to systematically manipulate body-weight support and stabilization-related mechanical demands while directly measuring the steady-state energetic responses. By isolating these factors, we sought to determine whether either mechanical task – body-weight support or lateral stabilization – imposes the dominant energetic burden during walking in CP. Insights from these experiments will provide a foundation for the development of targeted, energy-focused interventions that address the underlying biomechanical source of elevated energetic demand. This study represents a critical step toward explaining why walking is so energetically demanding for individuals with CP.

## Methods

### Study Design

This prospective, cross-sectional, laboratory-controlled experimental study addressed two primary aims: (1) to quantify the energetic cost of vertical body-weight support during walking, and (2) to quantify the energetic cost of mediolateral stabilization during walking. To address these aims, we had participants complete a series of treadmill walking trials under systematically varied levels of externally applied vertical body-weight support or mediolateral stabilization forces using a custom mechatronic system. The order of conditions was randomized. We measured the energetic cost of walking at each condition via indirect calorimetry and used the energetic cost at each condition to determine the relative effect of body-weight support and lateral stabilization tasks on overall energetic cost of walking.

### Study Sample

We recruited 24 children with bilateral CP, aged 8–17 years, classified as Gross Motor Functional Classification System (GMFCS) levels I, II, or III, representing all levels of ambulatory function. In addition, we recruited 10 TD peers stratified by age with equal representation of male and female participants (3M/3F per 2-year age group).

Our sample size estimates were based on differences in energetic costs observed from retrospective analyses of CP and TD energetic data at our center as well as previously reported changes in energetic cost of walking with body-weight support and external stabilization^52^. This resulted in a sample size estimate of 21 children with CP and 7 TD peers (3:1 allocation) using α = 0.05 and 1-β = 0.9.

We excluded individuals who had undergone orthopedic surgery within the past 12 months, received anti-spasticity injections within the past 2 months, were currently using oral or implanted anti-spasticity medications, or were unable to reliably follow instructions. We obtained informed consent and assent from all individuals prior to participation. The University of Washington Institutional Review Board approved all procedures (STUDY00014591), and the study was registered to ClinicalTrials.gov (NCT04303078).

### Experimental Protocol

#### Experimental Protocol

Participants completed treadmill walking trials under either the body-weight support protocol, the lateral stabilization protocol, or both when feasible. Each protocol consisted of a series of four externally applied force conditions while participants walked at a fixed speed. The order of protocols and the sequence of conditions within each protocol were randomized. Participants walked for 5 minutes at each condition to allow metabolic rate to stabilize, and we provided opportunities for rest between each condition, as needed. Additionally, we gave a minimum of 10 minutes of rest between body-weight support and lateral stabilization protocols. The total walking time per participant was 20 minutes per protocol (i.e., 40 minutes total when both protocols were completed).

We had participants perform all walking trials at a fixed nondimensional (ND) walking speed of 0.26 (approximately 0.7 m/s for an average child), which accounted for differences in leg length and ensured a consistent task demand across participants (e.g. similar passive leg swing dynamics). We calculated ND speed as:

$$\upsilon_{ND}=\upsilon /(\sqrt{gL})$$

Where *υ* is the walking speed (m/s), *g* is gravitational acceleration [m/s^2^], and *L* is leg length [m]. The selected speed corresponded to the 36th percentile of self-selected speeds among ambulatory children with CP (GMFCS Level I-III) previously evaluated at our center, ensuring that the majority of potential participants could complete the protocol at the fixed experimental speed.

### Aim 1: Vertical Body-weight Support

To isolate the energetic cost of body-weight support during walking, we applied external vertical forces to the pelvis using a custom mechatronic system (Humotech Inc., Pittsburgh, PA). The system delivered constant vertical support through a rotary magnetic motor and Bowden cable transmission, with an inline force transducer providing real-time force feedback to a custom controller. The actuator assembly was mounted to a two-degree-of-freedom ceiling-mounted gantry that allowed free horizontal motion within the experimental area. Horizontal translation was constrained to the treadmill walking surface, and vertical translation was limited to ± 6 inches from the participant’s static standing position. A modified climbing harness distributed the applied forces evenly across the pelvis.

For the first three participants, support levels were set at 1%, 25%, 50%, and 62.5% of body weight. These values were subsequently refined to 1%, 20%, 40%, and 60% of body weight for all remaining participants to achieve more uniform spacing across support conditions. An analysis of the system performance is provided in the ‘Body-weight Support System Performance’ section.

### Aim 2: Lateral Stabilization

To isolate the energetic cost of mediolateral stabilization during walking, we applied external lateral forces using the same mechatronic system, configured to provide spring-like stabilization and reduce participants’ need to actively control their center of mass. The system delivered mediolateral forces through a custom two-degree-of-freedom floor-mounted rail that allowed free fore–aft motion and adjustable height. A transmission cable attached to one side of the pelvis was paired with a spring bank on the opposite side, and a custom pelvic harness distributed forces while allowing free sagittal-plane rotation and modest coronal-plane motion. Inline force transducers measured net pelvic forces, which the real-time controller used to modulate applied forces based on pelvic displacement from the treadmill centerline and the current stiffness condition.

Unlike passive linear springs, we implemented an asymmetric stiffness controller that generated higher centering forces when participants moved away from the treadmill centerline and reduced forces when returning toward center. This strategy minimized oscillatory “bounce-back” behavior following lateral displacement.

For the first three participants, stiffness levels were set at 400, 800, 1200, and 1600 N/m. These values were subsequently refined to 50, 500, 1000, and 1500 N/m so that the lowest condition approximated a near-zero stiffness baseline. System performance is described in the ‘Lateral Stabilization System Performance’ section.

### Energetic Measurements

We quantified energetic cost using indirect calorimetry (CPX Ultim; Medgraphics, St. Paul, MN, USA). The system recorded breath-by-breath oxygen consumption (${\dot{\text{V}}\text{O}}_{2}$, mL O_2_/min), which we converted to energetic cost (J/s) using a caloric equivalent of oxygen of 20.1 J/mL O_2_, divided by 60. We then computed the net nondimensional (NN) energetic cost of walking as:

$${\text{Cost}}_{NN}=\frac{{\text{Cost}}^{\text{walking}}-{\text{Cost}}^{\text{sitting}}}{mg\sqrt{gL}}$$

where *Cost*^*walking*^ is the gross energetic cost of walking [J/s], *Cost*^*sitting*^ is the gross energetic cost of sitting [J/s], *m* is body mass [kg], *g* is gravitational acceleration [m/s^2^], and *L* is leg length [m]. We derived both *Cost*^*walking*^ and *Cost*^*sitting*^ from measured ${\dot{\text{V}}\text{O}}_{2}$ values.

**Figure 1:**
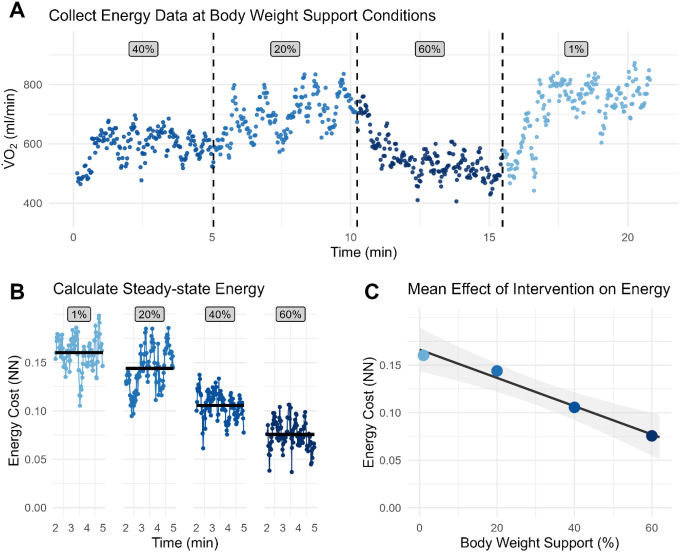
Example of data processing pipeline for a single representative participant. (**A**) Participant completed 5-minute walking trials under four randomized support conditions from 1%, 20%, 40%, and 60% body-weight support (shown here without breaks between conditions). (**B**) We calculated the net energetic cost for each sampled breath and then calculated the steady-state energetic cost for each condition as the mean of the final 3 minutes of walking data. (**C**) The overall intervention effect was evaluated using the slope of a linear model relating net energetic cost to the level of support.

We measured *Cost*^*sitting*^ during a 5-minute period while participants sat quietly in a chair to establish baseline energetic activity. We measured *Cost*^*walking*^ during a continuous 5-minute period for each walking condition (Fig. 1A). We calculated the average of the final three minutes of data from each trial to quantify steady state standing and walking energetic costs (Fig. 1B). We then used linear regression between NN energetic cost of walking and applied support or stiffness level (Fig. 1C) to analyze the effect size.

### Outcome Measures

The primary outcome was the slope of the linear regression between NN energetic cost of walking and applied support or stiffness magnitude (Fig. 1C). Negative slopes indicate reductions in energetic cost with increasing support or stabilization. Effect sizes were defined by the extrapolated difference between energetic cost at 100% body-weight support and at 1% body-weight support divided by the mean energetic cost of a TD control group walking at 0.26 ND speed without intervention. Effect magnitudes greater than 100% of the mean TD control value were categorized as ‘Large’, between 25% and 100% as ‘Small’, and less than or equal to 25% as ‘None’.

### Data Analysis

We tested our primary hypothesis – that support and stabilization tasks are key contributors to elevated energetic cost of walking in individuals with CP – by fitting linear regression models of NN energetic cost of walking versus support or stabilization level magnitude, respectively. The slopes of these regressions quantified the strength of this relationship, with effect sizes compared between CP and TD groups as well as reported individually.

To further examine how patient characteristics correlated with the effect of body-weight support and lateral stabilization on energetic cost of walking, we used a multiple regression model including age, sex, group (CP or TD), GMFCS level, baseline energetic cost, ND step width, mean knee flexion during stance, and BMI.

### System Validation

#### Body-weight Support System Performance

**Figure 2:**
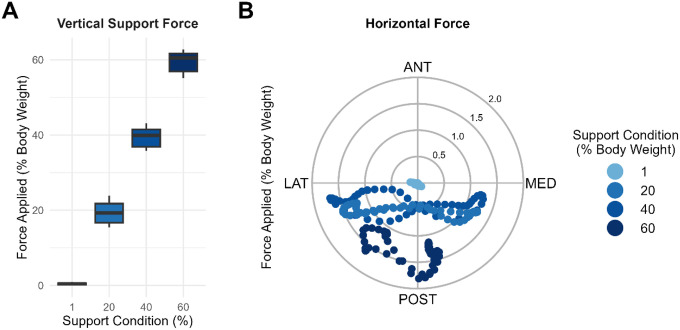
Vertical body-weight support system performance as a percent of body weight for a single gait cycle of a representative participant weighing 37.1 kg. (A) Vertical forces were applied at the desired body-weight support conditions. (B) Transverse-plane forces were substantially lower than the vertical support forces.

We verified the body-weight support system provided consistent vertical support forces during gait while minimizing unintended forces in other directions (Fig. 2). Mean vertical support errors were consistent across the 20%, 40%, and 60% conditions (−1.9 ± 6.9 N) with slightly lower variability at the 1% condition (−0.5 ± 4.7 N). This resulted in actual percent support conditions of 0.8 ± 0.9%, 19.6 ± 1.8%, 39.6 ± 1.8%, and 59.6 ± 1.9% for the 1%, 20%, 40%, and 60% targets, respectively. Performance was similar for the different initial experimental conditions used for the first three participants.

#### Lateral Stabilization System Performance

**Figure 3:**
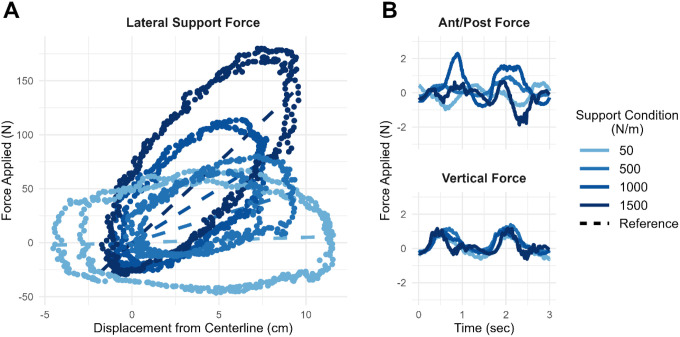
Performance of the lateral stabilization system for a representative participant purposefully displacing the pelvis approximately 10 cm from the treadmill centerline. (A) The asymmetric lateral force profile formed elliptical trajectories in a clockwise pattern, characterized by greater forces when moving away from the centerline and reduced forces when returning, thereby mitigating bounce back effects. (B) Frontal-plane forces were markedly smaller in magnitude than the applied lateral forces. Note: the magnitude of the displacement exceeds that observed during participant walking.

We also validated that the lateral stabilization system produced the intended medio-lateral support forces during gait while minimizing vertical and anterior–posterior forces. The force–stiffness controller delivered asymmetric forces depending on pelvic motion. When the pelvis moved away from the treadmill centerline, resistance increased according to the stiffness condition plus an asymmetric component. Conversely, when the pelvis moved toward the centerline, applied forces reduced accordingly. This control strategy prevented the development of a “bounce back” effect when the pelvis displaced from the centerline. We performed this validation with a representative participant purposefully displacing the pelvis approximately 10 cm from the treadmill centerline to demonstrate the system performance (Fig. 3).

Across stiffness conditions (50, 500, 1000, 1500 N/m), mean errors were consistent and minimal: − 0.1 ± 0.6 N in the anterior–posterior direction and 0.2 ± 0.5 N in the vertical direction, confirming that the system delivered accurate lateral forces with minimal unintended forces in other directions.

The energetic contributions of the anti–bounce-back control feature of the stabilization system were negligible. The median energy absorbed by the system was 0.4 J per gait cycle (IQR: 0.2–0.9) in the CP group and 0.3 J (IQR: 0.2–0.5) in the TD group, equivalent to 0.5% of gross energetic cost of walking in both groups, assuming 25% metabolic efficiency for positive work (IQR: 0.3–1.0% for CP and IQR: 0.4–1.0% for TD). Energy absorption was consistent across stiffness levels, driven largely by changes in pelvic motion, averaging 2.0 ± 1.0 cm in both groups at the lowest stiffness and decreasing to 1.8 ± 1.0 cm for CP and 1.4 ± 1.0 cm for TD at the highest stiffness condition.

## Results

### Participant Characteristics

A total of 23 children with CP and 10 TD peers completed the study protocol (Table 1). Age distributions were similar across CP and TD cohorts, and sex representation was balanced within the TD group (5:5) and moderately male-skewed in the CP group (15:8). Participants with CP had functional walking abilities across all ambulatory GMFCS levels: GMFCS I - Walks without limitations, GMFCS II - Walks with some limitations, and GMFCS III - Walks using assistive devices. Most participants with CP were classified as GMFCS II, consistent with the distribution observed in our historical gait laboratory population.

All 23 participants with CP completed the body-weight support protocol, while 14 completed the lateral stabilization protocol. All 10 TD participants completed both protocols. One additional CP participant consented to participate in the study but withdrew prior to completing either protocol due to behavioral challenges.

### Effect of Body-weight Support on Energetic Cost of Walking

**Figure 4:**
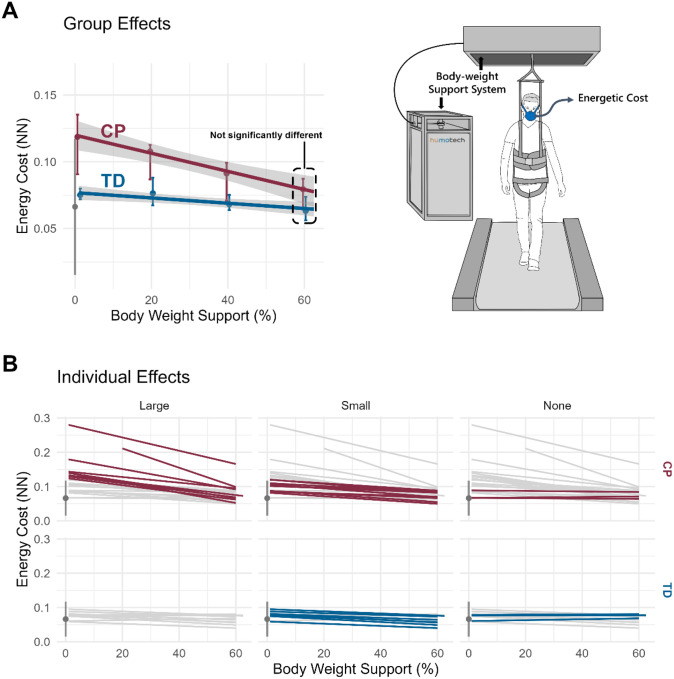
Effect of vertical support on energetic cost of walking for CP and TD participants. (**A**) Group averages and (**B**) individual responses categorized by effect size: large, small, or none. The gray vertical bar and point on the left of each plot represents the 90% CI and mean value for typically developing controls with no body-weight support at 0.26 ND walking speed. Colored error bars represent the IQR of responses for each group. Individual effect sizes were defined by the extrapolated participant response at 100% body-weight support minus the 1% support response divided by the mean control magnitude (gray point): >100% (Large), >25% (Small), or ≤25% (None). Note: y-axis scale is different between (A) and (B).

Providing body-weight support reduced the energetic cost of walking for both CP and TD groups. However, the effect was 3.5 times greater in the CP group (Fig. 4A). Energetic cost decreased by 6.78 × 10 − 4 per percent body-weight support in CP, compared to 1.98 × 10 − 4 in TD. This drop corresponds to a 41.1% vs 16.8% decrease in NN energetic cost across the full 1–60% support range for CP and TD, respectively. At 60% body-weight support, energetic cost of walking in CP was statistically indistinguishable from that of TD participants (p = 0.15), effectively normalizing the energetic cost of walking for this group.

Individual responses to body-weight support varied considerably (Fig. 4B). Linear regression analyses revealed strong participant-level linear relationships between energetic cost and support magnitude (r^2^ = 0.74 ± 0.29 in CP; 0.67 ± 0.37 in TD; reported as mean ± SD). Participants with higher baseline energetic cost exhibited larger reductions in energetic cost, indicating that benefit scales with baseline energetic demand.

Multiple linear regression analysis revealed that both higher baseline energetic cost and greater mean knee flexion during stance (i.e., crouch gait) predicted larger reductions in energetic cost (p < 0.001 and p = 0.002, respectively). No other demographic or gait variables – age, sex, GMFCS, step width, or BMI – were found to be significant predictors of changes in energetic cost (all p > 0.16).

### Effect of Lateral Stabilization on Energetic Cost of Walking

**Figure 5:**
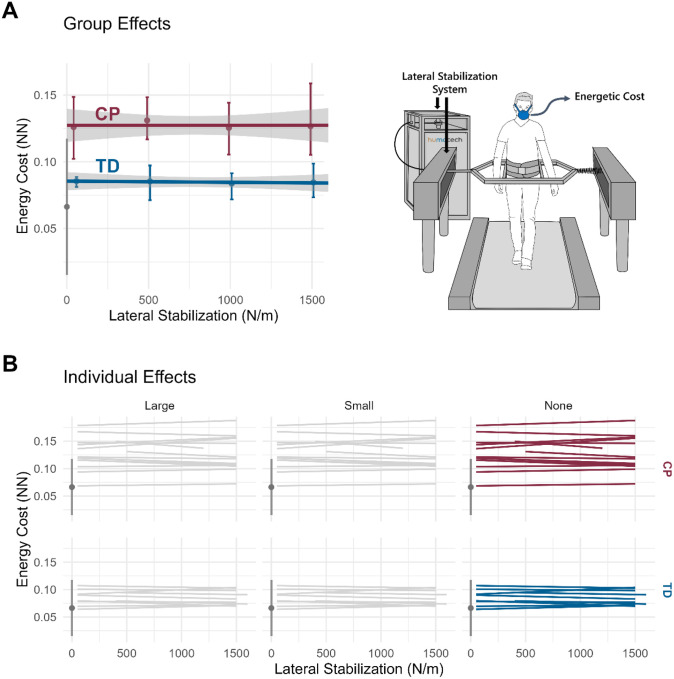
Effect of lateral support on energetic cost of walking for CP and TD participants. (**A**) Group averages and (**B**) individual responses categorized by effect size: large, small, or none. The gray vertical bar and point on the left of each plot represents the 90% CI and mean value for typically developing controls with no body-weight support at 0.26 ND walking speed. Colored error bars represent the IQR of responses for each group. Individual effect sizes were defined by the extrapolated participant response at 100% body-weight support minus the 1% support response divided by the mean control magnitude (gray point): >100% (Large), >25% (Small), or ≤25% (None).

In contrast to body-weight support, external lateral stabilization produced minimal changes in the energetic cost of walking for both groups (Fig. 5A). Across stiffness levels ranging from 50 to 1500 N/m, NN energetic cost changed by only 0.1% in the CP group, and − 1.5% in the TD group. The relative magnitude of the stabilization effect was less than 1% of the corresponding body-weight support effect in both groups.

Individual responses to lateral stabilization were small (Fig. 5B), with weaker linear regression fits between energetic cost and stiffness magnitude (r^2^ = 0.38 ± 0.29 in CP, 0.25 ± 0.24 in TD). Regression analyses did not identify participant characteristics that predicted energetic response to external lateral stabilization forces.

## Discussion

This study demonstrates that the energetic cost of body-weight support is the dominant physiological factor contributing to elevated walking cost in CP. By directly manipulating mechanical demands during walking, we show that reducing the requirement to support body mass against gravity produces large, consistent reductions in energetic cost, whereas reducing lateral stabilization demands yields negligible energetic benefit. These findings provide a mechanistic explanation for why many conventional interventions have failed to reliably reduce the energetic cost of walking in CP.

**Figure 6:**
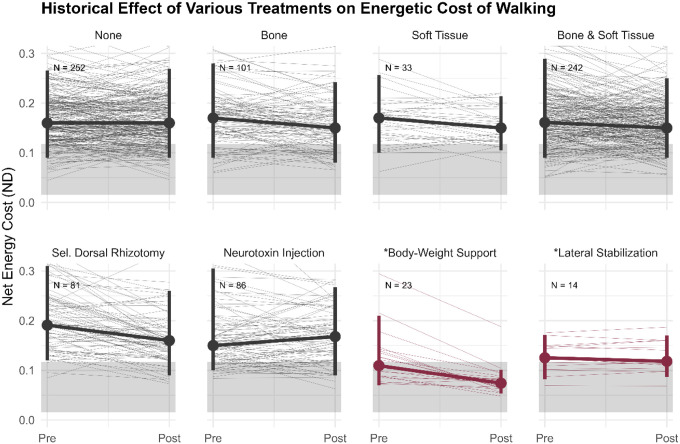
Changes in energetic cost of walking for common treatments groups compared to study interventions. Individuals with diplegic CP included in the historical group (black lines) were between 8–18 years of age at the Pre-visit, and time between the Pre- and Post-visit was less than 2 years. Individuals with diplegic CP in the study intervention group are shown in red. Vertical bars represent the 5^th^- 95^th^ percentile of energetic costs of walking, and the gray band represents the 90% CI for typically developing controls walking at 0.26 ND speed. *Note: Body-weight Support and Lateral Stabilization changes occurred during the same visit. Observed changes for the historical treatment groups (black) were not adjusted for covariates that could have influenced the results.

These findings are compelling in the context of decades of clinical experience and research. Historically, major interventions for children with CP, including orthopedic surgery to realign the skeleton, soft-tissue procedures to relieve contractures, selective dorsal rhizotomy and botulinum toxin injections to reduce spasticity, have not reliably reduced the energetic cost of walking^15,21,53–56^. When energetic benefits are reported (e.g., after multilevel surgery), they tend to be modest, variable, and highly dependent on study design, often lacking control groups for proper comparison^21,53,54^. Longitudinal data from our center mirror these findings, with inconsistent changes in energetic cost occurring over multi-year periods, which likely reflect developmental maturation rather than intervention-specific effects (Fig. 6). Notably, even the largest systematic reductions following selective dorsal rhizotomy resemble changes observed in a non-SDR control group, suggesting a developmental rather than intervention-specific origin.

In contrast, the results of this study demonstrate that providing body-weight support consistently and substantially reduces the energetic costs of walking in CP (Fig. 6). Participants with higher baseline energetic costs and more severe crouch gait experienced the greatest reductions in energetic cost, indicating that intervention benefit scales with the underlying biomechanical burden. These results align with prior work demonstrating that overall kinematic normality (e.g. knee-flexion angles during stance), and neuromuscular control (e.g. ability to selectively and dynamically control muscle timing and activations required for energy efficient gait) had the strongest causal influence on energetic cost of walking in CP^57,58^. Greater knee flexion during stance likely increases muscle activity required to support the center of mass against gravity, reducing reliance on passive skeletal alignment and elevating energetic costs^34^. While earlier studies reported only modest direct associations between crouch severity and energetic cost^57,59^, our findings indicate that knee flexion severity strongly influences responsiveness to interventions that target body-weight support.

The reductions in energetic cost of walking observed in TD participants are consistent with prior work for typically-developing adults showing that supporting and stabilizing the body against gravity during walking accounts for 10%−30% of the total energetic cost^25,26,52,60^. In this study, 60% body-weight support reduced gross energetic cost by 9.3% in TD participants, closely matching previously reported reductions in energetic cost of approximately 11% at 50% body-weight^60^. In contrast, even high levels of lateral stabilization (1500 N/m) produced minimal energetic effects, differing from earlier adult studies that reported modest 5.7% reductions in energetic cost with stabilization^25^.

### Limitations

**Figure 7:**
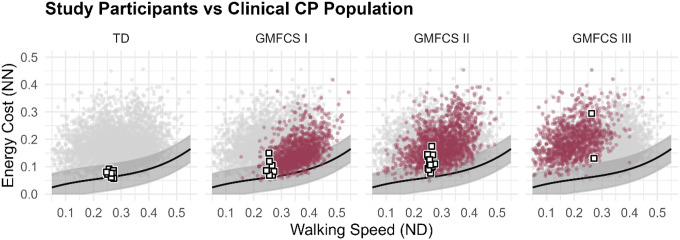
Distribution of study participants relative to the historical clinical CP population at our center. Of the participants in this study (white squares), the CP participants are biased toward lower energetic cost of walking compared to the historical CP dataset (red circles) at our center. The black line and shaded band represent mean±1SD values for typically developing controls. Note: The historical CP dataset is comprised of 4,012 analyses from 2,098 unique individuals.

Several limitations should be considered when interpreting these findings. The CP cohort was biased toward individuals with lower baseline energetic cost (Fig. 7). We suspect that the walking requirements (20-min per protocol) may have discouraged enrollment among individuals with the highest energetic burden. Standard steady-state energy estimation methods require ${\dot{\text{V}}\text{O}}_{2}$ to stabilize and be averaged over several minutes, typically requiring at least 5 minutes of walking per condition. We suspect that including individuals with higher energetic cost would likely accentuate the observed energetic benefits of body-weight support. Future studies should evaluate alternative steady-state energy estimation approaches that reduce the walking duration requirement (e.g. 3-min of walking per condition) to broaden participation^61^.

This experimental system required deliberate design choices that balanced feasibility with the complexity of human balance control and walking energetics. Delivering vertical body-weight support is relatively straightforward as it involved constant, mass-proportional force targets, whereas lateral stabilization required dynamic force modulation based on pelvic displacement. We implemented a linear stiffness controller for robustness and interpretability. However, the optimal paradigm for reducing balance-related energetic costs remains unknown. Alternative force control designs, such as nonlinear stiffness or adaptive controllers, may better reflect the demands of individuals with impaired stability which may result in different energetic effects than those observed in this study.

Finally, the fixed-speed walking paradigm simplified the complex interaction between speed, support, and walking energetics. Future work should examine variable walking speeds and speed-support interactions to better understand how support-targeted interventions translate to real-world outcomes in a broad range of walking environments.

### Translational and Clinical Implications

By identifying gravitational support as the primary driver of elevated energetic cost of walking in CP, this study provides a clear mechanistic target for intervention development. Interventions that target and reduce the mechanical and neuromuscular demands of body-weight support are likely to yield the largest energetic benefits. These approaches may include assistive technologies that offload partial body weight during rehabilitation or therapy, targeted strength training of antigravity muscle groups, surgical procedures that restore limb alignment and promote passive skeletal support, or emerging assistive technologies such as wearable exoskeletons to augment body-weight support capabilities^34,62–65^. Prior work demonstrating energetic reductions with exoskeleton assistance and body-weight supported treadmill training further supports this direction^63,65^.

In contrast, interventions that primarily target lateral stability are unlikely to substantially reduce energetic costs under conditions similar to those tested here. Nonetheless, interventions addressing lateral stability may still offer separate benefits for safety or function, emphasizing that energy optimization should complement, not replace, broader treatment goals.

Baseline walking cost strongly predicted responsiveness to body-weight support, suggesting that energetic cost could serve as a biomarker for treatment personalization and planning. This finding supports future concepts like energy-based phenotyping, in which baseline energetic measures are used to identify which patients are most likely to benefit from treatments targeting specific mechanical body-weight support deficits.

Overall, this work illustrates how controlled biomechanical experimentation can identify fundamental energetic constraints that shape mobility in CP. By isolating body-weight support as the dominant energetic contributor, these findings offer a mechanistic framework for designing targeted rehabilitation and assistive technologies aimed at improving walking efficiency, independence, and participation.

## Conclusion

Body-weight support is the dominant biomechanical contributor to elevated walking energetic cost in children with cerebral palsy, whereas lateral stabilization contributes minimally under the conditions tested. These findings identify gravitational support as a primary target for energy-focused rehabilitation and assistive technology interventions aimed at improving walking efficiency and participation.

## Figures and Tables

**Table 1 –:** Participant Demographics

Characteristic	CP N = 23	TD N = 10	p-value^1^
Sex			0.5
F	8 (35%)	5 (50%)	
M	15 (65%)	5 (50%)	
Age	12.8 (3.0)	12.8 (2.9)	> 0.9
Age Ranges			> 0.9
(8,10]	6 (26%)	2 (20%)	
(10,12]	4 (17%)	2 (20%)	
(12,14]	4 (17%)	2 (20%)	
(14,16]	6 (26%)	2 (20%)	
(16,18]	3 (13%)	2 (20%)	
GMFCS			N/A
I	7 (30%)	-	
II	13 (57%)	-	
III	3 (13%)	-	
N/A	-	10 (100%)	

Values reported as: n (%); Mean (SD)

1 Fisher’s exact test; Wilcoxon rank sum exact test

## Data Availability

Core data used in this study are available at: [https://clinicaltrials.gov/study/NCT04303078] (https://clinicaltrials.gov/study/NCT04303078)

## Abbreviations

CP: Cerebral palsy
TD: Typically developing
GMFCS: Gross Motor Functional Classification System
NN: Net nondimensional
ND: Nondimensional
BMI: Body mass index
${\dot{V}O}_{2}$: Oxygen consumption
